# Bridging the Computational-Experimental Gap: Leveraging Large Language Model to Prioritize Alzheimer’s Therapeutics Based on Comparison of Learning Models

**DOI:** 10.21203/rs.3.rs-7811754/v1

**Published:** 2025-11-07

**Authors:** Manqi Li, Shuteng Niu, Yifeng Xu, Jianfu Li, Xinyue Hu, Duan Liu, Merve Atik, Xiaolei Xu, Liewei Wang, Nilüfer Ertekin-Taner, Cui Tao

**Affiliations:** 1. Department of Biostatistics and Data Science, School of Public Health, The University of Texas Health Science Center at Houston, Houston, TX, USA.; 2. McWilliams School of Biomedical Informatics, The University of Texas Health Science Center at Houston, Houston, TX, USA.; 3. Department of Artificial Intelligence and Informatics, Mayo Clinic, Jacksonville, FL, USA.; 4. Department of Electrical and Computer Engineering, University of Washington, Seattle, WA, USA.; 5. Department of Molecular Pharmacology and Experimental Therapeutics, Mayo Clinic, Rochester, MN, USA.; 6. Department of Neuroscience, Department of Neurology, Mayo Clinic, Jacksonville, FL, USA.; 7. Department of Biochemistry and Molecular Biology, Department of Cardiovascular Medicine, Mayo Clinic, Rochester, MN, USA.

## Abstract

Alzheimer’s Disease (AD)^1^ is a progressive neurodegenerative disorder with limited therapeutic options, driving interest in drug repurposing to accelerate treatment discovery. Drug repurposing has emerged as a promising strategy to accelerate therapeutic discovery by repositioning existing drugs for new clinical indications. Recent computational repurposing approaches, including knowledge graph reasoning, transcriptomic signature analysis, and integrative literature mining, have demonstrated strong predictive capabilities^2^. However, these methods often yield divergent drug rankings, which makes it difficult to decide which candidates to advance for experimental follow-up and results in substantial gaps between computational predictions and feasible in vivo validation^2^.

To bridge this computational-experimental gap, we proposed an advanced prioritization framework leveraging large language models (LLMs). Our method systematically evaluated three state-of-the-art (SOTA) and representative computational methods (TxGNN^3^, Composition-based Graph Convolutional Network (CompGCN)^4^, and a regularized logistic regression (RLR)^5^, to analyze both their predictive performance and pharmaceutical class distributions. By integrating the strengths and divergences of these models, we generated a unified, streamlined list of 90 candidate drugs for further prioritization. We then utilized an LLM-based agent to perform evidence synthesis from biomedical literature abstracts for each candidate. This process mimics expert manual curation but significantly reduces human effort and time by efficiently distilling vast textual data into actionable insights. Applying consistent and transparent selection criteria, we obtained a refined and prioritized list of drug candidates suitable for subsequent in vivo experimental validation.

The robustness and clinical relevance of our framework were validated using real-world data from Alzheimer’s patient cohorts, clinical trial registries, and expert pharmacological reviews. This comprehensive validation confirmed that our LLM-driven approach enhances efficiency, consistency, scalability, and generalizability. By integrating computational predictions with scalable evidence synthesis and multifaceted validation, our framework facilitated rapid and informed prioritization of repurposed drugs. Our framework can potentially accelerate the translational pathway toward viable AD therapeutics. Moreover, the versatility of our framework can also be applied to drug repurposing efforts for other diseases beyond AD.

## Introduction

AD is a progressive neurodegenerative disorder characterized by memory loss, cognitive decline, and behavioral disturbances, and it remains a leading cause of disability among the elderly^1^. Despite decades of research, the available therapeutic options for AD are limited in both efficacy and scope, which has driven the biomedical community to explore alternative strategies for treatment development^2^. One promising approach is drug repurposing, a strategy that seeks to identify new therapeutic applications for existing FDA–approved compounds^6^. This method offers a significant advantage by bypassing many early-phase drug development challenges, including the time and financial costs of bringing a new drug to market^6^. As such, drug repurposing has garnered increasing attention as a viable, pragmatic pathway for addressing the urgent need for effective AD treatments.

In recent years, advances in Artificial Intelligence (AI) and big data have reshaped the landscape of drug repurposing^7,8^. Traditional databases and expert systems cannot meet the demands of modern biomedical knowledge discovery^2^. As an effective solution, Knowledge graphs (KGs) have become a key data representation for high volume and complex biomedical knowledge^9–12^. Accordingly, Graph Neural Networks (GNN)-based models have been adopted to perform downstream tasks, such as KG reasoning^13,14^, transcriptomic signature analysis^15^, and integrative data mining from biomedical literature^11^. In particular, KG completion has emerged as a promising approach for drug repurposing for AD^13,16^. Additionally, such innovative methods have improved the quality and efficiency of drug repurposing by mining deep hidden relations in biomedical concepts without labor-based literature mining. While the SOTA GNN models have made promising progress on drug repurposing, there are several limitations: 1) the validation process remains a manual and human-centered task, resulting in poor efficiency, high costs, and a lack of scalability^3,16^, 2)different learning models show significantly varying prediction behaviors corresponding different biomedical KG characteristics^17^, and 3) the methods often lack the generalizability to be readily applied across different diseases without significant retraining.

Moreover, traditional validation methods involve expert reviews, manual literature curation, and wet-lab experiments in the standard drug repurposing framework. While these methods are effective in cases with small and manually-manageable data, they suffer from a few unique limitations in big data, such as high cost, insufficient expert input, and incomplete literature coverage. The validation has become the new bottleneck of the computational drug repurposing framework, which prevents professionals from releasing the full potential of high-volume data and computational tools^18^. Furthermore, despite powerful Natural Language Processing (NLP) tools and standard ontologies, existing biomedical KGs have inherent sparse connections, imbalanced node types and relation types, as well as inaccurate knowledge^9^. Recently, various advanced graph models have been proposed to address these fundamental issues. However, different types of graph models demonstrate highly variant advantages and shortcomings^10^ (details and insights provided in the section Discussion Model Divergence). Firstly, traditional statistical models (logistic regression, random forest) with manually-designed graph features are not suitable for complex graphs and multi-relation^19^. Secondly, regular GNN models mine deeper patterns and show better robustness to noise from KG curation, but their performance is compromised by sparse connections^20^. Lastly, GNNs equipped with node similarity matching^20^ improve node representation with sparse connections, but the drug recommendations are often less accurate in AD. To date, there is no efficient and scalable way of harmonizing the outputs of different models to produce a single, reliable, and actionable list of candidates for a given disease.

To overcome these limitations, we proposed a novel LLM-based Alzheimer’s Disease Drug Repurposing (ADDR) framework, which integrated the strengths of multiple graph models and leverages the reasoning capabilities of LLM to create an efficient, automated, and scalable pipeline. Our framework was designed to tackle three central questions. First, we investigated why different graph models produced divergent predictions for AD by conducting a comprehensive analysis of their architectural principles and output distributions. Second, we established how to effectively validate and prioritize these divergent predictions by developing an LLM-driven module that aggregated evidence from multiple sources, mitigating the risks of model hallucination. Finally, we demonstrated how to efficiently verify the computational findings by corroborating our top-ranked candidates against Real-World Data (RWD), including an Electronic Health Record cohort and clinical trial data, as well as expert pharmacological reviews. In this final step, expert validation codified a reusable human-in-the-loop framework for drug prioritization.

To these ends, we developed simple yet effective solutions to all challenges. Our investigation started with a comprehensive evaluation of the graph models from both an algorithmic and a data-driven, domain-knowledge perspective. We turned to the data and domain-knowledge facets by examining the criteria used for feature selection and analyzed how different pharmaceutical classes were represented in each model’s predictions. Through this two-pronged analysis, we uncovered shared patterns and key divergences across models that directly informed the design of our LLM-prioritization strategy. Specifically, we examined three SOTA ADDR models (RLR, CompGCN, and TxGNN) end-to-end, from their core design principles through to their output distributions. The methodological insights gained from this analysis directly informed the design of our LLM-powered framework to automate the validation and prioritization processes. This system intelligently aggregates and ranks drug candidates by synthesizing evidence from multiple sources. To confirm its efficacy, we benchmarked our top candidates against real-world outcomes from the NACC Alzheimer’s cohort and clinical trial data. In parallel, we conducted a structured pharmacological review in which two independent clinicians systematically scored leading candidates across safety, mechanism of action, therapeutic breadth, and preclinical evidence, providing a rigorous human-expertise benchmark for the LLM’s prioritization. The real-world applicability of our integrated methodology was ultimately demonstrated as several drugs prioritized by the LLM were subsequently stratified through expert pharmaceutical review, proving our framework can accelerate the transition from in silico modeling to viable therapeutic discovery.

The remainder of the paper is organized as follows: Results demonstrates key findings of the proposed framework, including prioritized and validated therapeutic candidates; Discussion presents technical insights into model scalability, generalization capabilities, and outlines future research directions; Data sources and preparation describes the data sources and preprocessing steps; Methodology details the model architecture and implementation; and References and Supplementary materials includes additional experimental results, implementation details, and supporting resources.

## Result

**Figure 1.**
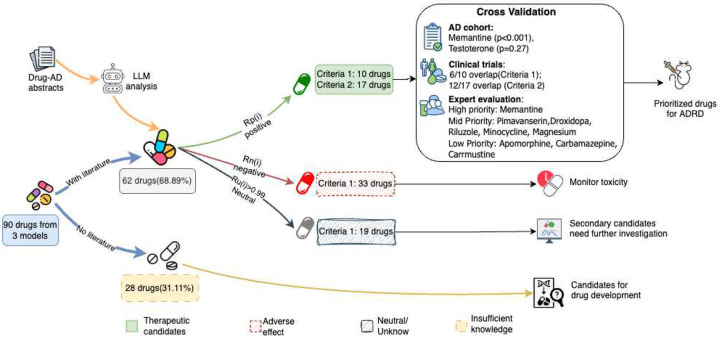
Strategy Framework of LLM-based Prioritization for AD: This diagram illustrated our three-stage workflow for narrowing down Alzheimer’s drug candidates. First, we aggregated 90 top-ranked compounds from three distinct graph-based models and retrieved all relevant PubMed abstracts. Next, an LLM processed each abstract and assigned one of three judgments (positive, neutral, or negative) to every drug–disease pair. Finally, we applied two complementary decision criteria to these aggregated judgments: a conservative filter that deferred classification when evidence was overwhelmingly neutral and otherwise selected the dominant signal, and a sensitivity-focused rule that flagged any hint of positive evidence. The resulting stratification highlighted high-confidence therapeutics, broader candidates meriting follow-up, compounds with adverse signals, and unexplored opportunities lacking published data. For details of the criteria design, please see the Methodology LLM-Based Prioritization Framework Section.

Our unified LLM-based framework systematically and automatically prioritized repurposable drugs for AD, starting from 90 candidates proposed by three SOTA models (TxGNN^3^, CompGCN^4^, and RLR^5^ on PrimeKG^21^). By leveraging the GPT-4.1 API^22^, we processed all 2,330 PubMed abstracts in approximately 1 hour and 11 minutes, yielding interpretable judgments of positive $\left(P_{i}\right)$, neutral $\left(U_{i}\right)$, and negative $\left(N_{i}\right)$ evidence for each candidate. As demonstrated in Figure 1, 62 out of 90 candidates were evidenced by AD-related literature and further evaluated using two evidence-based criteria (see Method: ADDR Framework). As shown in Table 1, under the stringent Criterion 1 (favoring a strong positive literature signal), 10 high-confidence candidates emerged, including both known and novel compounds (e.g., memantine, riluzole, minocycline, magnesium, and carmustine). A broader Criterion 2 identified 17 compounds with potential therapeutic relevance. Validation against a real-world AD patient cohort confirmed a statistically significant protective effect for memantine (p<0.001) and a suggestive trend for testosterone (p=0.27), prioritized under Criterion 1 and 2 respectively. Furthermore, our method recapitulated 6 of 28 compounds previously evaluated in clinical trials under Criterion 1, and 12 under Criterion 2, demonstrating substantial alignment with existing translational efforts. Importantly, 28 candidates (31.1%) lacked any AD-related publications, which underscores both the novelty of LLM-discovered candidates and the current knowledge gap, suggesting promising directions for preclinical exploration. To this end, two domain experts scored prioritized compounds using a four-criterion rubric (efficacy, safety, mechanism, and indication breadth). Memantine emerged as the only “high-priority” candidate (13/16), consistent with its NMDA-antagonist mechanism and extensive clinical validation. Five additional compounds (pimavanserin, droxidopa, riluzole, minocycline, and magnesium) were classified as “mid-priority” (>8/16), reflecting strong preclinical support and safety profiles, yet lacking fully elucidated mechanisms in AD.

To draw a conclusion, our results demonstrate that LLMs can effectively bridge computational predictions with human-interpretable biomedical evidence, revealing both validated and underexplored candidates for AD drug repurposing. The framework offers a scalable, literature-grounded framework for accelerating therapeutic discovery and prioritization across other disease domains.

### LLM-prioritized therapeutic candidates

We applied our unified LLM-based prioritization algorithm to the 90 candidate drugs identified by the three SOTA models (TxGNN^3^, CompGCN^4^, and RLR^5^, see Supplement materials: Preliminary work) on PrimeKG^21^. After retrieving AD-relevant PubMed abstracts for each drug, we obtained $T_{i}$ judgments per drug (partitioned into $P_{i}$ positive, $U_{i}$ neutral, and $N_{i}$ negative) and computed the rates $R_{p}(i)$, $R_{u}(i)$, and $R_{n}(i)$. Judgments were made on one abstract at a time (see Figure 1). Notably, 28 of these 90 candidates (31.11%) yielded no AD-related publications—most frequently among TxGNN predictions (13/30), then CompGCN (8/30), and finally RLR (7/30). This lack of literature evidence highlights a critical knowledge gap and indicates that these compounds may offer novel repurposing opportunities deserving experimental validation in vitro or in vivo.

#### Categorizing drug-AD relationships with LLM analysis

After excluding 28 compounds lacking AD-relevant literature, the remaining 62 candidate drugs were subjected to LLM-based evaluation using two criteria based on the statistical rates $R_{p}$ (positive), $R_{u}$ (neutral), and $R_{n}$ (negative).

Under Criterion 1, which deferred classification when neutral responses dominate ${(R}_{u}\geq 0.99)$ and otherwise assigned labels based on whether $R_{p}$ exceeded $R_{n}$, 10 drugs were identified as potentially therapeutic. These included istradefylline, pimavanserin, droxidopa, and apomorphine (from TxGNN); carbamazepine, memantine, riluzole, minocycline, and magnesium (from CompGCN); and carmustine (from RLR). In contrast, Criterion 2, which adopted a more permissive rule by flagging any compound with $R_{p}>0$, yielded a broader set of 17 potentially therapeutic candidates. In addition to those identified by Criterion 1, this set included chlorpromazine (TxGNN); dextromethorphan (CompGCN); and testosterone, naproxen, doxycycline, histamine, and paclitaxel (RLR). These drugs constituted a prioritized shortlist for experimental validation and follow-up in drug repurposing framework.

The evaluation further stratified compounds with ambiguous or negative literature profiles following Criterion 1. We identified nineteen drugs with highly neutral sentiment distributions ${(R}_{u}\geq 0.99)$, reflecting either insufficient investigation or lack of definitive conclusions in the literature. These compounds warrant targeted reviews or meta-analyses to resolve their standing in the AD therapeutic landscape.

Finally, thirty-three drugs were classified as potentially adverse under Criterion 1 ($R_{n}>R_{p}$ when $R_{u}<0.99$), indicating a preponderance of negative evidence or safety concerns. These compounds should be deprioritized for AD repurposing and considered for further toxicological and mechanistic scrutiny prior to any future translational efforts.

#### Divergent candidate rankings across learning models

The distribution of prioritized therapeutic candidates varied substantially across the three learning models. As shown in Table 1, under the stringent Criterion 1, CompGCN contributed half of therapeutics (5/10); whereas under the broader Criterion 2, all three models contributed similarly (CompGCN: 6/17; RLR: 6/17; TxGNN: 5/17). This divergence suggests the complementary strengths of different network-based learning approaches and the importance of ensemble-style integration for comprehensive drug repurposing frameworks. In response, our framework extracts candidate drugs from graph-based prediction models, retrieves relevant PubMed abstracts, and reasons abstract-level sentiment using an LLM-enabled systematic prioritization of repurposing candidates for ADDR.

In summary, 62 of the 90 initial candidates were supported by AD-related literature. Of these, 10 met Criterion 1 for high-confidence therapeutic potential, 17 satisfied the broader Criterion 2, 19 were literature-neutral, and 33 showed potentially adverse signals. This clear stratification refined via LLM-based analysis, demonstrated our framework’s ability to transform diverse model outputs into a transparent and experimentally-actionable shortlist.

### Validation against real-world clinical and trial data

To test the effectiveness of our LLM-based prioritization, we first cross-validated 90 candidate compounds against an AD patient cohort (hazard ratios with p-values were shown in Table 1) and then quantified its presence in AD-related trials by investigating registered clinical trials (counts and IDs of trials were shown in Table 1).

We first evaluated our LLM-based prioritization against an AD patient cohort. As the drugs in bold (Table 1) show, among the drugs exhibiting a statistically significant protective effect, the LLM correctly recovered memantine (p<0.001) as a potential therapeutic under both Criterion 1 and Criterion 2. Of four drugs with moderate cohort-level signals (zolpidem (p=0.29), dexamethasone (p=0.24), prednisolone (p=0.14), and testosterone (p=0.27)), only testosterone was prioritized under Criterion 2. Remarkably, the model also flagged magnesium and carmustine as candidate therapeutics under both criteria despite no supporting evidence in the AD cohort, underscoring the LLM’s capacity to surface plausible repurposing opportunities beyond those directly observed in clinical data. While the LLM appears somewhat more conservative than cohort analysis in recovering known signals, neither approach serves as a definitive gold standard: cohort associations may be confounded by unmeasured factors or limited by small sample sizes. Nonetheless, the concordance between cohort-derived associations and LLM predictions supports the utility of our LLM-prioritized framework for efficiently narrowing the search space of AD drug repurposing candidates.

Next, we assessed the 90 candidate drugs against AD-related clinical trial records (Table 1 Clinical Trial Count column). Among the candidates, twenty-eight drugs had at least one registered trial. Under Criterion 1, the LLM identified 6 of these trial-tested drugs as potential therapeutics; under Criterion 2, it recovered 12 of the 28. Using clinical trial inclusion as a proxy for prior expert endorsement, these results demonstrate that our LLM-based method can reproducibly highlight historically recognized candidates in a conservative manner, mirroring its performance in the cohort cross-validation. Moreover, of the 10 drugs flagged under Criterion 1, 6 overlapped with clinical trial compounds (leaving istradefylline, droxidopa, carbamazepine, and carmustine as novel predictions), and of the 17 drugs flagged under Criterion 2, 12 had trial records (leaving paclitaxel novel). These “new” candidates illustrate the model’s ability to leverage existing biomedical knowledge and propose fresh hypotheses for future AD drug-repurposing efforts.

Taken together, the cohort and trial validations demonstrate that the LLM-based framework can recover known therapeutic signals and propose candidates overlooked by existing evidence sources.

### Expert pharmacological review for efficacy of prioritized candidates

To benchmark the LLM-based prioritization against human expertise, two independent clinicians scored 10 candidate therapeutics (those consistently highlighted by both criteria) across four domains: preclinical effectiveness, safety and tolerability, mechanism of action, and therapeutic breadth (0–4 points each, maximum 16; details were shown in Supplement File 1). This exercise was designed to stratify LLM-prioritized agents, not to validate the framework’s global performance (no negative-control drugs were included).

Memantine ranked highest by both approaches. As shown in Table 1, it met both LLM criteria and showed significant cohort protection (HR = 0.59, p < 0.001) and the largest AD trial footprint (97 trials). Expert scores (13/16 from both reviewers; mean = 13) were concordant with this status, citing robust preclinical/clinical evidence, favorable tolerability, and a well-defined NMDA-antagonist mechanism.

Pimavanserin, minocycline, magnesium, riluzole, and droxidopa were all classified as “potentially therapeutic” under LLM criteria, though trial and cohort support varied. Expert scoring placed these in the intermediate range (mean 8–9). Pimavanserin (mean 9) received solid safety and mechanistic ratings, consistent with LLM prioritization and its presence in four AD trials. Minocycline (mean 9) and magnesium (mean 9) were similarly reinforced by expert consensus, though magnesium lacked cohort signal (no HR available), highlighting the LLM’s role in surfacing candidates outside limited real-world datasets. Riluzole (mean = 8.5) and droxidopa (mean = 8) both exhibited stronger preclinical efficacy than breadth of action, which the LLM captured as therapeutic potential but with modest trial representation.

Istradefylline, apomorphine, carbamazepine, and carmustine scored 6–6.5 on average, reflecting safety concerns, limited breadth, or unclear AD-relevant mechanisms. Notably, the LLM still classified each as “potentially therapeutic”, despite a lack of clinical trial activity for most (except apomorphine). This suggests that while the LLM effectively detects literature-level positive signals, expert domain knowledge remains critical for contextualizing translational feasibility and safety.

Within the LLM-selected set, expert review largely re-ordered and refined priorities rather than validating the framework in an absolute sense. Concordance at the top (e.g., memantine) and mid-tier alignment suggest that the LLM’s literature-level signals track key expert considerations, while expert adjudication adds penalties for safety liabilities and narrow mechanisms that are less evident from abstract-level text. This post-LLM triage step mirrors a human-in-the-loop workflow and is readily reusable across other domains and disease areas.

## Discussion

In this study, we introduced a novel framework to bridge the persistent gap between high-throughput computational drug repurposing and practical experimental validation for AD. Our results demonstrate that by systematically reconciling the divergent outputs of multiple SOTA models and leveraging the evidence-synthesis capabilities of an LLM, we can produce a transparent, prioritized, and clinically relevant list of therapeutic candidates. This discussion will first interpret the underlying reasons for the observed model divergence, then evaluate the effectiveness, scalability, and generalizability of our proposed framework, and finally consider its current limitations and future potential.

### Model divergence reveals complementary predictive strengths

A central challenge in computational drug repurposing is that models with comparable predictive performance often yield divergent candidate lists. Our analysis of TxGNN, CompGCN, and an RLR based on DWPC confirms this phenomenon and, more importantly, reveals that this divergence stems directly from their distinct architectures and feature representations.

TxGNN emphasizes disease-similarity embeddings to produce a “low-risk” shortlist of repurposing candidates whose approved indications cluster around neurodegenerative disorders topologically proximal to AD. More than half of TxGNN’s top 30 predictions target diseases such as Parkinson’s and other nervous-system disorders (Supplement Figure 1, Table 1), which may expedite clinical translation by leveraging established safety profiles. The tradeoff, however, lies in a potentially narrower search space that privileges well-characterized disease networks at the expense of uncovering less explored mechanisms^23^.

CompGCN’s edge-composition and node-aggregation message-passing framework embeds both node and relation semantics, propagating information through densely connected subgraphs. Consequently, it uncovers a remarkably broad array of pharmacological classes, including cholinesterase modulators, antineoplastics, neuropsychiatric agents, and latent links such as memantine, despite the absence of explicit indication edges (Supplementary Figure 2, Supplementary Table 2). This diversity makes CompGCN well-suited for exploratory hypothesis generation. Yet, operating on an undirected KG without explicit semantic constraints can lead to misattribution: for example, phenobarbital, an anticholinergic with mechanisms opposed to existing cholinergic AD therapies (donepezil, rivastigmine, and galantamine)^24^, was elevated despite counter-therapeutic mechanisms. Mitigating such semantic conflation may require integrating fine-grained relation-type modeling or attention-based supervision.

RLR based on DWPC transforms manually defined metapaths into interpretable graph features, favoring anti-inflammatory corticosteroids (Supplementary Figure 3, Supplementary Table 3) that align with epidemiological evidence of inflammation’s role in AD^25^. Its transparent, interpretable design readily surfaces known drug–phenotype associations, making RLR an efficient tool for phenotype-driven candidate validation. However, its reliance on shallow, predefined four-hop metapaths limits discovery of deeper or entirely novel pathways.

These distinct profiles underscore that there is no single “best” model; instead, their strengths are complementary. The choice of model can be tailored to specific strategic goals: TxGNN for safety-oriented repurposing, CompGCN for broad mechanistic exploration, and RLR for hypothesis confirmation based on known phenotypes.

### Framework effectiveness: from model divergence to actionable insight

The inherent divergence among these models, while offering complementary perspectives, creates a significant challenge for downstream prioritization. Our LLM-driven framework was designed specifically to address this by harmonizing heterogeneous outputs through scalable, evidence-based curation. Its effectiveness was validated through retrospective cohort analysis and cross-referencing with registered clinical trials.

In our analysis of a real-world AD patient cohort, the framework demonstrated a balance of specificity and sensitivity. It correctly prioritized memantine (p<0.001), a drug with a robust protective signal, under both its stringent (Criterion 1) and permissive (Criterion 2) rules. For drugs with weaker cohort signals like testosterone (p=0.27), it selectively recovered them under the more sensitive Criterion 2, showcasing the utility of the dual-threshold design. Critically, the framework also identified candidates like magnesium, which, despite lacking a significant signal in our cohort data, was rated as a “mid-priority” compound in the subsequent expert evaluation. This illustrates the LLM’s capacity to surface plausible candidates that retrospective observational data alone might overlook.

Cross-validation against clinical trial data further confirmed the framework’s fidelity. It successfully recovered a substantial portion of previously tested drugs (6/28 under Criterion 1, 12/28 under Criterion 2), affirming its ability to reproduce expert-endorsed hypotheses. More importantly, it generated high-confidence, novel predictions such as droxidopa and carmustine, which have no prior AD trial record but were deemed plausible by our framework and, in the case of droxidopa, received a “mid-priority” expert rating. This dual capability to both confirms existing evidence and generate novel, high-potential leads underscores its value as both a validation tool and an engine for discovery.

### Scalability and generalizability: a versatile discovery platform

A primary bottleneck in drug repurposing is the manual, labor-intensive process of evidence reviews. Our framework automates and accelerates this step. The retrieval and classification of 2,330 PubMed abstracts for 90 candidates were completed in just 1 hour and 11 minutes—a task that would typically demand weeks of expert effort. This efficiency demonstrates that the framework can scale with a growing volume of candidates and literature without prohibitive resource demands, making rapid, literature-grounded hypothesis generation feasible across diverse therapeutic areas.

Crucially, we pair automation with an expert-in-the-loop triage step. After LLM prioritization, two independent clinicians scored the shortlisted agents to refine ordering based on translational considerations (e.g., preclinical effectiveness, safety/tolerability, mechanism, breadth). This post-LLM review mirrors a human-in-the-loop framework that is easy to reuse in other domains and diseases, adding a lightweight layer of clinical judgment without re-introducing the full burden of manual curation. Together, automated literature synthesis and targeted expert adjudication deliver a scalable, generalizable platform that balances discovery breadth with translational plausibility.

### Limitations and future directions

Despite its promising performance, our framework has several limitations that open avenues for future refinement.

First, the quality and structure of the underlying KG are paramount. Sparse or undirected relationships can bias predictions, as seen with CompGCN’s mis-ranking of phenobarbital. Future work should focus on incorporating more granular, directed relation types and supervision to mitigate such semantic ambiguities.

Second, our literature analysis relies on PubMed abstracts, which may not capture the full context available in full-text articles or account for publication bias^26^. Furthermore, some novel predictions lacked any literature, highlighting a knowledge gap. To probe the reliability of our LLM’s abstract classifications, we performed a manual validation on a subset of 50 abstracts, revealing an 82.00% agreement rate with human experts. The primary sources of disagreement were twofold: 1) the LLM acted conservatively on preclinical (animal or cell) studies, often classifying them as ‘neutral’ where an expert would see a ‘positive’ signal, and 2) the LLM sometimes classified abstracts as ‘negative’ when drug-AD keywords co-occurred without a stated relationship, whereas an expert would deem this ‘neutral’. This latter issue suggests opportunities for refinement through prompt engineering. Expanding the evidence base to include full-text articles, RWD from electronic health records, and multi-omic datasets would enhance the robustness and depth of the evidence synthesis.

Third, the framework’s reasoning is dependent on the pre-training knowledge of LLM. While we opted for a constrained, non-web approach using curated corpora to ensure reproducibility and avoid hallucination, performance could be enhanced by fine-tuning models on domain-specific biomedical literature.

Looking forward, we envision a dynamic, iterative discovery ecosystem. The knowledge graph could be continuously updated with emerging biological data. A virtuous cycle could be established where the top in-silico predictions are validated in preclinical models (e.g., organoids), with the experimental results feeding back to refine both the graph models and the LLM classifier. This closed-loop approach would progressively enhance predictive accuracy and accelerate the translation of computational insights into tangible clinical impact.

### Conclusion

Computational drug repurposing for complex illnesses like Alzheimer’s Disease is frequently hampered by predictive models that produce divergent and overwhelming lists of candidates, creating a significant bottleneck for experimental validation and clinical translation. In this work, we have demonstrated a novel hybrid framework that successfully overcomes this challenge. By integrating the outputs of complementary graph-based algorithms and leveraging a Large Language Model for rapid, automated evidence synthesis, we transformed a wide array of computational predictions into a single, prioritized list of therapeutic candidates.

Our approach was validated through real-world patient data, clinical trial records, and expert pharmacological review, confirming its ability to identify both established treatments and promising new candidates. The framework’s true strength lies in its capacity to harmonize high-throughput in silico screening with deep, contextualized evidence appraisal in a manner that is transparent, scalable, and reproducible. This methodology not only accelerates the discovery of viable therapeutics for AD but also provides a powerful and generalizable blueprint for bridging the critical gap between computational prediction and clinical application across a spectrum of human diseases.

## Data sources and preparation

### Biomedical KG selection

PrimeKG is a comprehensive biomedical KG comprising 129,375 nodes across 10 entity types and 4,050,249 edges across 33 relation types^21^. It integrates data from 20 publicly available resources to enable rich and multi-domain connectivity, including DisGeNET, DrugBank, SIDER, Gene Ontology, Reactome, UBERON, etc. Compared with existing networks such as Hetionet^27^, iBKH^11^, and PharmKG^28^, PrimeKG is particularly adept at reasoning about repurposable drugs due to its significantly more extensive drug-drug relation(33.0%). PrimeKG categorizes drug-disease relations into three types: ‘Drug_contraindication_Disease’, ‘Drug_indication_Disease’, and ‘Drug_off-label use_Disease’. This unique classification facilitates machine learning and deep learning efforts to explore drug-disease connectivity with different semantic meanings. Our study focuses on predicting the “indication” relation between AD and each of the 7,957 drug nodes in PrimeKG.

### Complementary pharmaceutical corpora for LLM analysis

To leverage the summation and classification capabilities of LLM, we assembled two complementary corpus, pharmacological descriptions from DrugBank(https://go.drugbank.com)^29^ and drug–AD abstracts from PubMed^30^. DrugBank has served as a gold-standard repository of drug information since 2006, offering detailed annotations of drug targets, pharmacodynamics, and chemical categories. For each of the 7,957 PrimeKG drug entities, we extracted the “Description”, “Pharmacology” (indication, pharmacodynamics, mechanism of action, protein binding), and “Category” fields. This textual metadata enriches the purely structural information in PrimeKG, mitigating the limitations of graph-only representations.

PubMed (https://pubmed.ncbi.nlm.nih.gov/about/) provides access to MEDLINE^31^ and contains over 38 million biomedical citations. We queried PubMed using the names and synonyms (from DrugBank and MeSH) of the top 90 candidates predicted by three ADDR models, in conjunction with “Alzheimer Disease.” Limiting retrieval to abstracts published from 2000 onward and capping at 200 abstracts per query, we collected a total of 2,330 abstracts. These texts supply context on reported drug–AD associations and serve as input for the LLM’s evidence-synthesis framework.

### External validation resource

We leveraged both Alzheimer’s cohorts dataset and clinical-trial registries to externally validate our LLM-based prioritization.

First, we obtained the Uniform Data Set (UDS) from the National Alzheimer’s Coordinating Center (NACC)^32^, one of the largest, longest-running Alzheimer’s cohorts, on June 17, 2024. The dataset comprises 188,701 longitudinal visit records through the March 2024 data freeze. We fitted a left-truncated, right-censored Cox proportional hazards model to investigate the drug efficacy. The detailed methodology is elaborated in the Methodology Statistical Analysis of the NACC Alzheimer’s Cohorts part.

Second, we queried ClinicalTrials.gov (https://clinicaltrials.gov/) for AD–related drug trials and cross-referenced these against our LLM-prioritized list. By triangulating real-world outcomes from NACC with prospective evidence from registered trials, this multi-faceted approach demonstrates the robustness of our LLM validation and highlights candidates already evaluated in human studies for further investigation.

## Methodology

**Figure 2:**
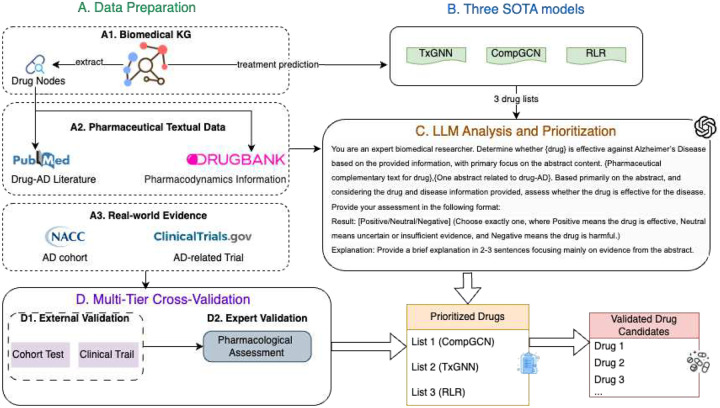
Framework Graph of LLM-based Prioritization for AD Therapeutics: The framework operates in four stages: (A) Diverse data is aggregated from a biomedical KG(PrimeKG), textual sources (PubMed, DrugBank), and real-world evidence (NACC, ClinicalTrials.gov). (B) Three SOTA models generate initial candidate drug lists. (C) An LLM analyzes these candidates by synthesizing evidence from the textual data to create a prioritized list. (D) Finally, these drugs undergo multi-tier cross-validation using external data (NACC, ClinicalTrials.gov) and expert pharmacological review to produce a final list of validated candidates.

The methodology for this study is organized into four principal stages: data preparation, candidate generation, LLM-driven prioritization, and multi-tier validation. A detailed schematic of this framework is presented in Figure 2, which outlines the flow from data aggregation to the final validated drug candidates.

### Problem definition

The central problem this research addresses is the systematic prioritization of existing drugs for repurposing as AD therapeutics. This involves bridging the gap between divergent computational predictions and actionable experimental validation. We formulate this as a multi-stage process involving knowledge graph-based prediction, LLM-driven evidence synthesis, and multi-faceted evaluation.

Let G=(𝒱,ε) be a biomedical KG whose nodes include drug nodes ${𝒱}_{\text{drug}}\subseteq 𝒱$ and disease nodes ${𝒱}_{\text{disease}}\subseteq 𝒱$. Given a target disease $d^{*}\in {𝒱}_{\text{disease}}$ and a biomedical corpus $T={\left\{\left(dr_{i},t_{i}\right)\right\}}_{i=1}^{n}$, where $dr_{i}\in {𝒱}_{\text{drug}}$ is a candidate drug and $t_{i}$ is the set of textual evidence for drug candidates $dr_{i}$, the objective is to generate a prioritized and stratified list of drug candidates for treating $d^{*}$. We employ m graph models that predict the probability that each $dr_{i}$ treats $d^{*}$:

$$f_{j}\left(dr_{i}|d^{*},G\right)\in \left[0,1\right],j=1,\ldots ,m.$$

For each model, let $L_{j}$ denote the top-ranked predictions under a threshold. The candidate pool is the union:

$$L=\cup_{j=1}^{m}L_{j}.$$

To aggregate textual evidence, an LLM–based labeling function, $\phi \left(d^{*},t_{i}\right)\in \{+1,0,-1\}$ maps each drug’s textual evidence $t_{i}$ to a relevance label (positive, neutral, negative) with respect to $d^{*}$. A universal prioritization function $\psi \left(dr_{i},f_{j}\left(dr_{i}|d^{*},G\right),\phi \left(d^{*},t_{i}\right)\right)\in \{0,1\}$ determines whether $dr_{i}$ should be included in the prioritized list. The final output is a stratified collection of validated drug candidates:

$$\hat{D\left(d^{*}\right)}=\left\{dr_{i}\in L|\psi \left(dr_{i},\cdot ,\cdot \right)=1,V_{\text{ext}}\left(dr_{i},d^{*}\right)=1,V_{\text{exp}}\left(dr_{i},d^{*}\right)=1\right\},$$

where $V_{\text{ext}}$ and $V_{\text{exp}}$ denote validation functions using external evidence (e.g., medical cohort for $d^{*}$, trials) and expert assessment, respectively.

### ADDR Framework

The LLM-based framework was executed within the Google Colaboratory environment on a virtual machine equipped with an NVIDIA T4 GPU and 16 GB of GDDR6 memory. The analysis of all PubMed abstracts was completed in approximately 1 hour and 11 minutes. To generate an initial candidate set, we first evaluated three SOTA models on the PrimeKG knowledge graph for AD indication prediction. We extracted the top 30 ranked candidates from each model, yielding a consolidated set of 90 unique drugs. For each drug–AD pair, we retrieved the relevant abstracts and utilized a structured prompt (see Figure 2) for an LLM (GPT4.1 API) to classify the relationship between drug and AD in each abstract as “positive”, “neutral”, or “negative”. For each drug $d_{i}$, the LLM produced a set of judgements from a total of $T_{i}$ abstracts, which can be partitioned into positive $\left(P_{i}\right)$, neutral $\left(U_{i}\right)$, and negative $\left(N_{i}\right)$. From these, we defined three rates to quantify the drug’s overall evidence profile:

$$R_{p}(i)=\frac{P_{i}}{T_{i}},R_{u}(i)=\frac{U_{i}}{T_{i}},R_{n}(i)=\frac{N_{i}}{T_{i}}$$

where $T_{i}=P_{i}+U_{i}+N_{i}$ and consequently $R_{p}(i)+R_{u}(i)+R_{n}(i)=1$. These rates form the basis of our unified ranking algorithm, ensuring each candidate is assessed by the same quantitative criterion.

Two distinct criteria were defined to accommodate different decision-making needs. Criterion 1 was designed to identify candidates with a clear signal by first filtering out those with overwhelmingly inconclusive evidence. Criterion 2 was designed to maximize sensitivity, flagging any candidate with even a single piece of positive evidence. Formally, for each drug for each drug $d_{i}$:

$${\text{Criterion}}_{1}(i)=\left\{\begin{array}{c} \text{Neutral}\;\text{Relation}\;R_{u}\left(i\right)\geq 0.99,\\ \text{Potentially}\;\text{Therapeutic}{\;R}_{u}\left(i\right)<0.99\wedge R_{p}(i)\geq R_{n}(i),\\ \text{Potentially}\;\text{Adverse}\;\text{Effect}{\;R}_{u}\left(i\right)<0.99\wedge R_{n}(i)\geq R_{p}(i), \end{array}\right.$$

$${\text{Criterion}}_{2}(i)=\left\{\begin{array}{ll} \text{Potentially}\;\text{Therapeutic} & R_{p}(i)>0,\\ \text{No}\;\text{Positive}\;\text{Sign} & \text{otherwise}. \end{array}\right.$$

Together, ${\text{Criterion}}_{1}$ captures nuanced, context-dependent judgments while ${\text{Criterion}}_{2}$ ensures that no candidate with even minimal positive evidence is overlooked.

### Statistical analysis of the NACC Alzheimer’s cohorts

The UDS from the NACC^32^ comprised 188,701 longitudinal visits from 50,962 participants. At baseline, the mean age was 71.18 ± 10.41(mean ± standard deviation (SD)) years and mean years of education were 15.85 ± 7.96 (mean ± SD); 57.19% were female and 42.81% male. At enrollment, marital status was distributed as 63.20% married, 16.06% widowed, 11.68% divorced, and the remainder separated, never married, cohabiting/domestic partner, or other. The living situation at enrollment was 61.63% with spouse/partner, 23.12% alone, 9.05% with a relative or friend, 4.00% in group residence, and the rest other or unknown. Cognitive status at enrollment was 40.39% normal cognition, 4.43% impaired-not-MCI, 22.30% MCI, and 32.88% dementia; at the end of follow-up, these proportions were 41.39%, 4.09%, 18.29%, and 36.22%, respectively. Drug exposures in the UDS were mapped to PrimeKG drugs via UMLS and Drugbank identifiers. To approximate chronic use, only drugs documented at two or more consecutive visits were included in the analysis.

To estimate each drug’s association with the time to cognitive decline, we fitted a Cox proportional hazards model. This semi-parametric model is ideally suited for this analysis as it accommodates time-to-event data characterized by left-truncation (participants entering the study at different ages) and right-censoring (participants leaving the study for reasons other than the event of interest, such as death or loss to follow-up), both of which are features of the NACC longitudinal cohort^33^. The model was defined as:

$$h\left(t|X_{i}\right)=h_{0}(t)\text{exp}\left(\beta_{\text{drug}}X_{i,\text{drug}}+\beta_{\text{age}}X_{i,\text{age}}+\beta_{\text{sex}}X_{i,\text{sex}}+\beta_{\text{edu}}X_{i,\text{edu}}+\beta_{\text{mar}}X_{i,\text{mar}}+\beta_{\text{live}}X_{i,\text{live}}\right)$$

where $h_{0}(t)$ is the baseline hazard function, the covariates $X_{i}$ included the binary indicator for chronic drug use as well as baseline age, sex, education, marital status, and living situation for participant i, and each β is the log-hazard coefficient for its covariate. Our analysis focused on the hazard ratio for the drug effect, $HR_{\text{drug}}=\text{exp}\left(\beta_{\text{drug}}\right)$, which quantifies the relative hazard of cognitive decline for exposed versus non-exposed participants. An $HR_{\text{drug}}>1$ indicates an increased risk (adverse effect), while an $HR_{\text{drug}}<1$ suggests a protective effect.

Statistical significance was evaluated at two distinct thresholds. We used the conventional alpha level of p<0.05 to declare a statistically significant association, as this standard minimizes the probability of a Type I error (a false positive)^34^. In addition, we considered a more relaxed threshold of p<0.30 for exploratory purposes, as noted in Table 1. This less stringent cutoff is often used in epidemiological and discovery-oriented research to identify potentially meaningful trends or signals that might be missed with a stricter threshold, thereby reducing the risk of Type II errors (false negatives) at the cost of including some false positives in a preliminary screening phase^35,36^.

## Supplementary Material

1

## Figures and Tables

**Table 1:** Potentially therapeutics prioritized by LLM with cross-validation

Drug Name	Graph Model	LLM-based Analysis					Hazard Ratio(p)	Clinical Trial Count (ID)
		$R_{u}(i)$	$R_{p}(i)$	$R_{n}(i)$	Criterion 1	Criterion 2		
Istradefylline	TxGNN	0.50	0.25	0.25	Potentially therapeutic	Potentially therapeutic	0.02 (0.95)	0
Chlorpromazine*	TxGNN	0.78	0.04	0.17	Potentially adverse effect	Potentially therapeutic	0.001 (0.93)	2(NCT02309723,NCT00975481)
Pimavanserin *	TxGNN	0.75	0.13	0.12	Potentially therapeutic	Potentially therapeutic	12.60 (0.01)	4(NCT02992132,NCT03325556,NCT03118947,NCT02035553)
Droxidopa	TxGNN	0.50	0.50	0	Potentially therapeutic	Potentially therapeutic	0.00 (0.90)	0
Apomorphine*	TxGNN	0.89	0.11	0	Potentially therapeutic	Potentially therapeutic	67.27 (<0.001)	2(NCT03837067,NCT04653584)
Carbamazepine	CompGC N	0.90	0.08	0.02	Potentially therapeutic	Potentially therapeutic	0.94 (0.82)	0
**Memantine** *	CompGC N	0.79	0.17	0.04	Potentially therapeutic	Potentially therapeutic	0.59 (<0.001)	**97**(NCT00545974,NCT01626391,NCT01409694,NCT00353665,NCT05669365)
Riluzole*	CompGC N	0.71	0.29	0	Potentially therapeutic	Potentially therapeutic	1.92 (0.19)	3(NCT00353665,NCT03605667,NCT01703117)
Dextromethorphan *	CompGC N	0.98	0.005	0.015	Potentially adverse effect	Potentially therapeutic	1.66 (0.18)	14(NCT02442765,NCT01584440,NCT00726726,NCT00056524,NCT01832350)
Minocycline*	CompGC N	0.67	0.25	0.08	Potentially therapeutic	Potentially therapeutic	1.24 (0.57)	1(NCT01463384)
Magnesium*	CompGC N	0.97	0.02	0.01	Potentially therapeutic	Potentially therapeutic	NA	18(NCT04656860,NCT05728229,NCT00490568,NCT04606420,NCT04251182)
Carmustine	RLR	0.50	0.50	0	Potentially therapeutic	Potentially therapeutic	NA	0
**Testosterone** *	RLR	0.90	0.03	0.07	Potentially adverse effect	Potentially therapeutic	0.84 (0.27)	**5**(NCT00392912,NCT02727699,NCT02018497,NCT00539305,NCT00000177)
Naproxen*	RLR	0.65	0.05	0.30	Potentially adverse effect	Potentially therapeutic	0.94 (0.38)	4(NCT00007189,NCT01417130,NCT02702817,NCT00004845)
Doxycycline*	RLR	0.84	0.04	0.12	Potentially adverse effect	Potentially therapeutic	0.95 (0.81)	4(NCT00439166,NCT04846335,NCT00715858,NCT00692588)
Histamine*	RLR	0.93	0.02	0.05	Potentially adverse effect	Potentially therapeutic	NA	8(NCT01268020,NCT01505504,NCT00987220,NCT06169826,NCT01009255)
Paclitaxel	RLR	0.88	0.02	0.10	Potentially adverse effect	Potentially therapeutic	0.01 (0.95)	0

Note: The drugs are listed in the order ranked within each graph model. Bold drugs were significant in the cohort (p<0.30) and prioritized by any Criteria of LLM;

“*” indicates drugs were tested in clinical trials and also prioritized by any criterion of LLM. We surveyed publicly registered trials on ClinicalTrials.gov (e.g., NCT02309723) related to the drugs predicted by our model to provide contextual evidence from prior literature. These trials were not part of our dataset or analyses, and no participants from these trials were enrolled or contacted.

## Data Availability

Data from NACC can be requested through https://naccdata.org/requesting-data/submit-data-request. Data from PrimeKG are available at https://dataverse.harvard.edu/dataset.xhtml?persistentld=doi:10.7910/DVN/IXA7BM. Other datasets generated and analyzed during the current study are available at https://github.com/maggielee1111/LLM-priotization-Framework/tree/main.

## Code availability

The underlying code and datasets for this study are available via this link https://github.com/maggielee1111/LLM-priotization-Framework/tree/main.
