# Supplementary material for: Bridging the Computational-Experimental Gap: Leveraging Large Language Model to Prioritize Alzheimer’s Therapeutics Based on Comparison of Learning Models
Source: Res Sq. 2025 Nov 7:rs.3.rs-7811754. Preprint. [Version 1] doi: 10.21203/rs.3.rs-7811754/v1 (PMC12637838; doi:10.21203/rs.3.rs-7811754/v1)
Supplement: 1 [file NIHPPRS7811754V1-supplement-1.pdf]

Supplement materials

Preliminary work: candidate generation models

We selected three SOTA drug-repurposing models: TxGNN, CompGCN, and RLR based on DWPC from the Hetionet Project. Each of these models demonstrated exceptional performance on its original benchmark: TxGNN achieved an AUPRC of 0.913, CompGCN an MRR of 0.355, and the RLR model an AUROC of 0.974. Although evaluated on different datasets and metrics, all three models exhibit high predictive accuracy in silico. We therefore applied them uniformly to the PrimeKG to predict AD indications and generate candidate drug lists for LLM-based prioritization. To better interpret their outputs, we next examined each model's feature representations and architectural design.

TxGNN

TxGNN is a graph model designed for zero-shot drug repurposing, enabling the identification of potential therapeutic candidates even for diseases lacking established treatments. It employs a

Graph Neural Network (GNN), a model that iteratively updates node representations by aggregating information from their neighbors, within a large-scale medical knowledge graph (PrimeKG). One of the cores about TxGNN is that it generates vector embeddings for drugs(e_{drug}) and diseases ($e_{disease}$) based on topological similarity.
The predictive model can be conceptualized as $y \sim f_{TxGNN}(e_{drug}, e_{disease})$.
In this formula, the outcome y is a predicted score indicating the likelihood that the drug is an indication for the disease. The predictor ($e_{drug}, e_{disease}$) are the learned vector embedding of the drug and disease. These embeddings are intermediate representations generated by the GNN encoder. Specifically, e_{drug} represents the learned latent vector embedding for the specific drug being queried. This embedding captures information about how the drug connects to targets within one hop, associated pathways, known indications/contraindications, genes, etc. Similarly, $e_{disease}$ represents the learned latent vector embedding for the specific disease being queried, which captures information about how the disease connects to targets within one hop. The overall TxGNN workflow progresses from processing the knowledge graph with the GNN encoder to obtain these embeddings, to using a metric learning decoder to predict the drug-disease relationship based on embedding similarity. This approach enables TxGNN to effectively learn from biomedical knowledge encoded in the graph structure, make drug repurposing predictions, even under zero-shot scenarios.

CompGCN

CompGCN is a framework designed to learn representations from multi-relational graphs, where nodes are connected by edges that have specific types (relations) and directions. Unlike traditional Graph Convolutional Networks (GCNs) that primarily handle simple, undirected graphs or struggle with the complexity and parameter explosion of numerous relation types, CompGCN learns vector embeddings for both the nodes (entities) and the relations simultaneously. It achieves this by incorporating composition operations (e.g., subtraction, multiplication, circular-correlation) inspired by KG embedding techniques. During the message passing phase, the representation for a target node is updated by aggregating information from its neighbors, where each neighbor's contribution is computed by composing the neighbor node's embedding with the embedding of the connecting relation using a chosen operator.

To manage the complexity and potential over-parameterization, CompGCN uses relation-type specific weight matrices (e.g., distinct weights for incoming, outgoing, and self-loop edges) and transforms relation embeddings alongside node embeddings across layers. This allows relation information to be shared and refined throughout the network. Furthermore, it offers a scalable approach for graphs with many relations by optionally using a basis decomposition technique, where initial relation embeddings are represented as linear combinations of a smaller, learnable set of basis vectors. This method generalizes several prior multi-relational GCN approaches and is shown to be effective for tasks requiring understanding of graph structure and relationships, such as link prediction.

To apply the CompGCN to predict treatment relation between drugs and diseases, we frame the prediction model as follow:

$y \sim f_{CompGCN}(h_{drug}, h_{relation}, h_{disease})$,
where y represents the score indicating the existence of a specific relation between a particular drug and a particular disease. The predictors $(h_{drug}, h_{relation}, h_{disease})$ are the features derived from the CompGCN's learned embeddings for specific drug, relation and disease. The model essentially generates these predictors as input features to get a scoring function $f_{CompGCN}$ to produce the outcome y. Based on the preliminary performance, we chose the ConvE as score function and circular-correlation as the composition operation.

RLR based on DWPC

RLR based on degree-weighted path count (DWPC) was developed in the Hetionet project (https://github.com/dhimmel/learn) to capture the meta - path topology of any drug–disease pair in the KG. A meta-path (λ) is a schema (e.g. Drug→Gene→Phenotype→Disease) describing a sequence of node types and edge types. For each meta-path λ , the DWPC between a source node s and target node t is defined as the sum over all path instances p of the product of each intermediate node's degree raised to the negative damping exponent $-w = -0.4$:

$$DWPC_{\lambda}(s,t) = \sum_{p \in P_{\lambda}(s,t)} \left\{ \prod_{v \in p \backslash \{s,t\}} deg(v)^{-w} \right\}$$

Here, $P_{\lambda}(s,t)$ is the set of all walks of type λ from s to t, $deg(v)$ is the degree of each intermediate node v, and w controls how strongly high-degree nodes are down weighted.

This formulation ensures that having many connecting walks increases the feature value, while walks through highly connected (less informative) nodes are down weighted. Each meta-path's DWPC then serves as a feature in an elastic-net-regularized logistic regression (via the glmnet package) that predicts the probability of a therapeutic relationship between the source drug and target disease; regularization both prevents overfitting across hundreds of meta-path features and induces sparsity in the learned coefficients. A RLR is trained on these DWPC features to predict the probability y of a therapeutic relationship:

$$Logit(y) = \beta_0 + \textstyle\sum_{\lambda} \beta_{\lambda} DWPC_{\lambda}(s,t).$$

Here, $\beta_0$is the intercept, and β represents the vector of coefficients for the meta path λ from source s to target t. In addition to DWPC features, the model incorporates a prior probability of indication derived from node-degree–based prevalence. Because the original Cypher-based feature extraction relied on network permutations and proved computationally intensive, we utilized the hetnet_ml framework (https://github.com/mmayers12/hetnet_ml) to accelerate DWPC computation in our treatment prediction on PrimeKG.

Supplementary Table 1. Chemical families of the top 30 candidates for AD in TxGNN

Drug Name	Chemical Family	Drug Name	Chemical Family	Drug Name	Chemical Family
Nifurtimox	Agents against leishmaniasis and trypanosomiasis (anti-parasite products, insecticides and repellents)	Opicapone	**Anti-parkinson agents (nervous system)**	Levacetylmethadol	**Drug for opioid dependence (nervous system)**
Deutetrabenazine	**Drugs for nervous system**	Cenegermin	Ophthalmological (sensory organs)	Dornase alfa	Drug for cough and cold preparations (respiratory system)
Levomethadone	**Drugs for nervous system**	Pimavanserin	**Antipsychotics (nervous system)**	Elexacaftor	Drugs in respiratory system
Tetrabenazine	**Adrenergics (nervous system)**	Vinpocetine	**Psychostimulants and nootropics (nervous system)**	Triflupromazine	**Antipsychotics (nervous system)**
Adenosine phosphate	Adenine Nucleotides (cardiovascular system)	Arformoterol	Adrenergics(respiratory system)	Dalfampridine	**Amines (nervous system)**
Istradefylline	**Anti-parkinson agents (nervous system)**	Alirocumab	Lipid modifying agents (cardiovascular system)	Tolcapone	**Anti-parkinson agents (nervous system)**
Piribedil	**Anti-parkinson agents (nervous system)**	Dihydroergocornine	**Drug for dementia and cognitive changes**	Clortermine	Amphetamines and derivatives
Safinamide	**Anti-parkinson agents (nervous system)**	Succinimide	Drug for genito urinary system and sex hormones	Pirfenidone	Immunosuppressant (antineoplastic and immunomodulating agents)
Valbenazine	**Drugs for nervous system**	Droxidopa	Adrenergic agonists (cardiovascular system)	Sivelestat	Hippuric acids (Drugs in respiratory system)
Chlorpromazine	**Antipsychotics (nervous system)**	Apomorphine	Drug for genito urinary system and sex hormones	Polidocanol	Anti-varicose therapy (cardiovascular system)

Supplementary Figure 1: Chemical families of the top 30 candidates for AD in TxGNN

(Nineteen chemical families; mean 1.6 drugs per family)

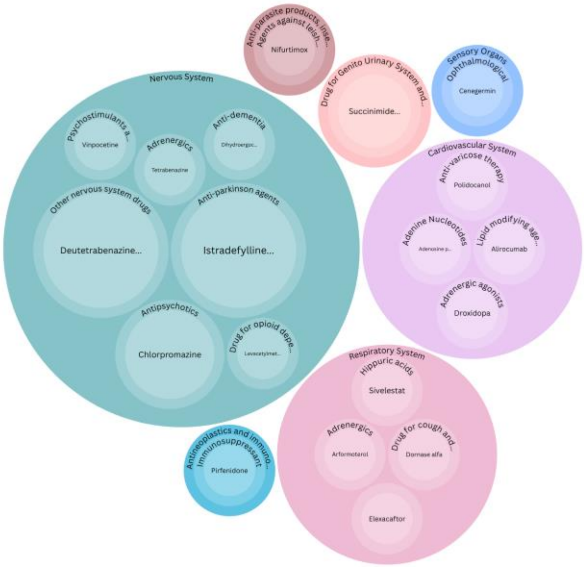

Supplementary Table 2. Chemical families of the top 30 candidates for AD in CompGCN

**Drug Name**	**Chemical Family**	**Drug Name**	**Chemical Family**	**Drug Name**	**Chemical Family**
Pyridostigmine	Anticholinesterases **(nervous system)**	Neostigmine	Anticholinesterases **(nervous system)**	Zolpidem	Psycholeptics **(nervous system)**
Prochlorperazine	Antipsychotics **(nervous system)**	Carbamazepine	Antiepileptics **(nervous system)**	Nizatidine	Acid Reducers (Alimentary tract and metabolism)
Tretamine	Antineoplastics and immunomodulating agents	Pimozide	Antipsychotics **(nervous system)**	Phenytoin	Antiepileptics **(nervous system)**
Phenobarbital	Antiepileptics; Anticholinergics **(nervous system)**	Memantine	Anti-dementia Drugs, Anticholinesterases **(nervous system)**	Acetophenazine	Antipsychotics **(nervous system)**
Thiotepa	Antineoplastics and immunomodulating agents	Paliperidone	Antipsychotics **(nervous system)**	Aprotinin	Antifibrinolytics (blood and blood forming organs)
Mechlorethamine	Antineoplastics and immunomodulating agents	Haloperidol	Antipsychotics **(nervous system)**	Perazine	Antipsychotics **(nervous system)**
Perphenazine	Antipsychotics **(nervous system)**	Procainamide	Antiarrhythmic (cardiovascular system)	Diethylcarbamazine	Antinematodals (anti-parasite products, insecticides and repellents)
Ropinirole	**Anti-parkinson agents (nervous system)**	Riluzole	**Other nervous system drugs**	Fluphenazine	Antipsychotics **(nervous system)**
Loxapine	Antipsychotics **(nervous system)**	Dextromethorphan	Antidepressive Agents Cough and cold preparations (**nervous system,** respiratory system)	Primidone	Antiepileptics **(nervous system)**
Alimemazine	Antihistamines (respiratory system)	Minocycline	Anti-infectives and antiseptics for local oral treatment; Anti-acne preparations; Anti-bacterials (Alimentary tract and	Magnesium	Mineral supplements (Alimentary tract and metabolism)

			metabolism; dermatologicals;antiinfectives for systemic use)		

Supplementary Figure 2: Chemical families of the top 30 candidates for AD in CompGCN

(Fifteen chemical families; mean 2 drugs per family)

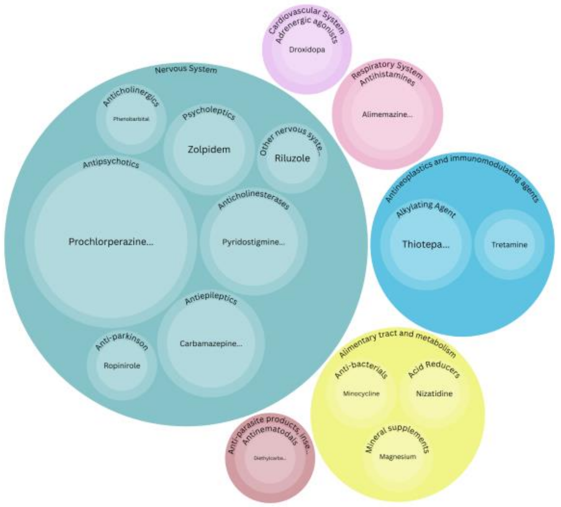

Supplementary Table 3. Chemical families of the top 30 candidates for AD in RLR based on DWPC

Drug Name	Chemical Family	Drug Name	Chemical Family	Drug Name	Chemical Family
Hydrocortisone	Corticosteroids; Anti-inflammation (alimentary tract and metabolism)	Thiotepa	Alkylating agents (antineoplastic and immunomodulating agents)	Testosterone	Drug for genito urinary system and sex hormones
Cortisone acetate	Corticosteroids; Anti-inflammation (alimentary tract and metabolism)	Fusidic acid	Medicated dressing with antiinfectives (dermatologicals)	Midostaurin	Antineoplastics
Hydrocortisone acetate	Corticosteroids; Anti-inflammation (alimentary tract and metabolism)	Carmustine	Alkylating agents (antineoplastic and immunomodulating agents)	Naproxen	Anti Inflammatory and antirheumatic products; Analgesics (nervous system, musculo-skeletal system,genito urinary system and sex hormones)
Dexamethasone	Corticosteroids; Anti-inflammation (alimentary tract and metabolism)	Belotecan	Plant alkaloids and other natural products (antineoplastic and immunomodulating agents)	Ofloxacin	Antibacterials (anti infectives)

Prednisolone	Corticosteroids;<br>Anti-inflammation<br>(alimentary tract and metabolism)	Benzylpenicillin	Antibacterials<br>(antiinfectives)	Doxycycline	Anti-infectives and antiseptics for local oral treatment;<br>Antibacterials<br>(alimentary tract and metabolism, antiinfectives)
Doxorubicin	Anthracyclines<br>(antineoplastic and immunomodulating agents)	Mechlorethamine	Antineoplastics	Adalimumab	Immunosuppressant<br>(antineoplastic and immunomodulating agents)
Triamcinolone	Corticosteroids;<br>Anti-inflammation<br>(alimentary tract and metabolism)	Norfloxacin	Antibacterials<br>(antiinfectives)	Histamine	Immunostimulants<br>(antineoplastic and immunomodulating agents)
Betamethasone	Corticosteroids;<br>Anti-inflammation<br>(alimentary tract and metabolism)	Olaparib	Antineoplastics	Benzatropine	**Anti-parkinson agents (nervous system)**
Prednisone	Corticosteroids;<br>Anti-inflammation<br>(alimentary tract and metabolism)	Promazine	Antipsychotics<br>**(nervous system)**	Procyclidine	**Anti-parkinson agents (nervous system)**
Methylprednisolone	Corticosteroids;<br>(alimentary tract and metabolism)	Tretamine	Antineoplastics	Paclitaxel	Antineoplastics

Supplementary Figure 3: Chemical families of the top 30 candidates for AD in RLR based on DWPC

(Thirteen chemical families; mean 2.2 drugs per family)

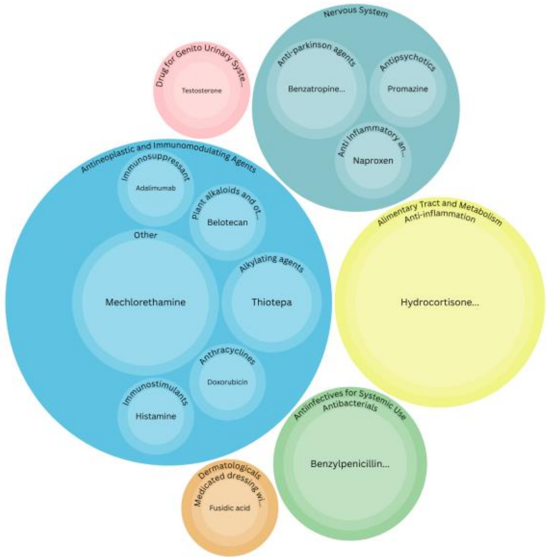

Supplementary Table 4. Expert Evaluation for 10 potential therapeutics

Drug	Preclinical Effectiveness (0-4)		Safety and Tolerability (0-4)		Mechanism of Action (0-4)		Therapeutic Breadth (0-4)		Total (0-16)		Average (0-16)
Istradefylline	2	2	0	3	2	1	1	2	5	8	6.5
**Pimavanserin**	2	**3**	3	3	2	2	1	2	**8**	**10**	**9**
**Droxidopa**	**3**	**3**	0	**4**	2	2	1	1	6	**10**	**8**
Apomorphine	2	**3**	0	3	1	2	1	1	4	9	6.5
Carbamazepine	2	2	1	2	2	1	**2**	1	7	6	6.5
**Memantine**	**3**	**3**	**4**	**4**	**4**	**3**	**2**	**3**	**13**	**13**	**13**
**Riluzole**	2	**3**	2	3	2	2	1	2	7	**10**	**8.5**
**Minocycline**	**3**	**3**	2	3	2	2	1	2	**8**	**10**	**9**
**Magnesium**	**3**	**3**	0	**4**	3	1	**2**	2	**8**	**10**	**9**
Carmustine	2	2	1	1	2	1	**2**	1	7	5	6

Supplementary File. 1 Evaluation Rubric for Assessing Alzheimer's Disease Drug Efficacy

Please use the following rubric to quantitatively assess the efficacy of each drug listed for Alzheimer's Disease (AD) treatment based on your expertise. Rate each category **from 0 to 4**:

Scoring scale:

- **0**: No evidence / Not effective
- **1**: Minimal evidence / Slightly effective
- **2**: Moderate evidence / Moderately effective
- **3**: Strong evidence / Highly effective
- **4**: Exceptional evidence / Extremely effective

Assessment Categories:

1. Preclinical Effectiveness

- Improvement in cognitive function in animal models
- Reduction in Alzheimer's pathology (e.g., amyloid plaques, tau phosphorylation)
- Restoration of neurotransmitter or biochemical markers

2. Safety and Tolerability

- Reported side effects or toxicity
- Acceptability of safety profile in both animal and human studies

3. Mechanism of Action

- Clear understanding of how the drug targets Alzheimer's pathology
- Evidence supporting the proposed biological mechanism

4. Therapeutic Breadth

- Drug’s ability to address multiple aspects of Alzheimer’s pathology (e.g., neuroinflammation, oxidative stress, amyloid clearance, cognitive symptoms)
